# Associations between Thermal and Physiological Responses of Human Body during Exercise

**DOI:** 10.3390/sports5040097

**Published:** 2017-12-19

**Authors:** Suleyman Zora, Gorkem Aybars Balci, Muzaffer Colakoglu, Tahsin Basaran

**Affiliations:** 1 Department of Energy Engineering, Izmir Institute of Technology, 35430 Izmir, Turkey; zorasuleyman@gmail.com; 2 School of Physical Education and Sports, Ege University, 35100 Izmir, Turkey; muzaffer.colakoglu@ege.edu.tr; 3 Department of Architecture, Izmir Institute of Technology, 35430 Izmir, Turkey; tahsinbasaran@iyte.edu.tr

**Keywords:** thermal comfort, predicted mean vote, exercise, ratings of perceived exertion

## Abstract

In this study, thermal behaviours of the athletes were investigated with respect to thermal comfort and exercise intensity. The relationship between an index for analysing thermal comfort (Predicted Mean Vote: PMV) and Rating of Perceived Exertion (RPE) which shows exercise intensity and exhaustion level was evaluated. Eleven moderately trained male athletes ($\dot{V}$O_2max_ 54 ± 9.9 mL∙min^−1^∙kg^−1^) had volunteered for the study (age: 22.2 ± 3.7 years; body mass: 73.8 ± 6.9 kg; height: 181 ± 6.3 cm; Body surface area (BSA): 1.93 ± 0.1 m^2^; body fat: 12.6% ± 4.2%; $\dot{V}$O_2max_: 54 ± 9.9 mL∙min^−1^∙kg^−1^). Experiments were carried out by using a cycle ergometer in an air-conditioned test chamber which provided fresh air and had the ability to control the temperature and relative humidity. The study cohort was divided into two groups according to maximal oxygen consumption levels of the participants. Statistical analyses were conducted with the whole study cohort as well as the two separated groups. There was a moderate correlation between PMV and RPE for whole cohort (*r*: −0.51). When the whole cohort divided as low and high aerobic power groups, an average correlation coefficient at high oxygen consumption cohort decreased to *r*: −0.21, while the average correlation coefficient at low oxygen consumption cohort increased to *r*: −0.77. In conclusion, PMV and RPE have a high correlation in less trained participants, but not in the more trained ones. The case may bring to mind that thermal distribution may be better in high aerobic power group in spite of high RPE and thus the relation between PMV and RPE is affected by exercise performance status.

## 1. Introduction

The human body is a dynamic system which is controlled and regulated by the brain. It has different behaviours at different metabolic loads and environmental conditions. Humans need permanent energy to continue their lives and vital functions. It is used not only in physical effort but also to maintain homoeothermic status with a core temperature of about 37 °C. Except for the mechanical energy for the physical effort, some of the energy can be stored in the body, the rest of which must be dissipated into the environment as heat. The lowest required energy is called the “basal metabolic rate” which is necessary to maintain basal metabolism. Basal metabolism depends on sex, age, and body fat rate [1]. The human body can easily adapt to alternating thermal conditions. Thermoregulation is closely related to thermal comfort. The heat balance between the environment and the human body must be equalized to provide thermal comfort. Generated energy in the metabolism should be equal to the heat loss from the body if there is no mechanical work. Besides, the core and skin temperatures are desired to be neutral for thermal comfort [2]. The thermoregulatory system is also the heat response of the body to the environment. There are two types of thermoregulations: behavioural and physiological [1]. Behavioural thermoregulation is related to the physical activity. For instance, putting on or taking off clothes, moving around and changing posture. Physiological thermoregulation is triggered by temperature signals from the core and the skin of the body. These signals are transmitted to the hypothalamus.

Nowadays, a lot of the people exercise regularly. During exercise, the human body adapts to the exercise conditions. The core body temperature rises depending on required energy, which obtains requirement via biochemical reactions in the human body. Only a limited amount of the energy transforms to mechanical energy, while the remaining part converts to thermal energy. One of the ways to protect the human body against excessive heat load by thermoregulatory system is sweating and dissipation. There is a thermal interaction between the human body and the environment during exercise and recovery periods of the exercise. With the thermal comfort expectation and the developed technology, the air conditioning system is applied not only to residences, offices and commercial buildings, but also the gymnasiums and sport halls. Similarly, sports economy is growing consistently with rising economic growth of sponsorship. In addition, a lot of people join sportive activities as audiences or participants compared to the past. Participating sportive activities as an audience or an athlete become one of the most important parts of daily life. On the other hand, exercise is one of the most important tools necessary for maintaining a healthy life.

Predicted Mean Vote (PMV), proposed by Fanger in 1970, is used frequently to evaluate the environmental conditions in terms of thermal load and sensation based on the steady state energy balance method. It estimates the mean response of a large number of humans with reference to the American Society of Heating, Refrigerating, and Air-Conditioning Engineers (ASHRAE) scale [3]. This scale ranges from +3 to −3 from hot to cold, and the other numbers are defined as; +2: Warm, +1: Slightly warm, −1: Slightly cool, and −2: Cool. Zero is the neutral value for the thermal comfort. Drifting from zero conceive warm and cold discomfort feelings and affects human performance and health. The numbers of thermal comfort studies on sports activities have been recently increasing and the thermal comfort conditions have been evaluated for athletes and audiences. A few of them focused on thermal comfort using PMV scale at an athletic competition or during an exercise [4,5]. These studies have focused on air conditioning specifications in athletic competition [4], heat interaction between the human body and the environment at different metabolic heat rate and different clothing insulations values [5]. According to experimental studies, PMV overestimated the thermal sensations of occupants in warm environmental conditions. A new empirical formula was generated for applying PMV to use for hot and humid climates [6]. Ye et al. carried out tests at actual and simulated environments with a new PMV measurement tool and compare the results with each other [7]. The tests were performed by using two different metabolic heat rate values as 1.0 Metabolic Equivalent of Task (MET) and 1.5 MET, and the operative temperature was approximately 25 °C. There was a similar tendency between 1.0 and 1.5 MET values at lower than 0.5 m/s air velocity value. At higher than 0.5 m/s air velocity, the difference between two MET values was not significant and it could be negligible. Kilic et al. investigated heat interactions between the human body and environment and calculated PMV individually [8]. The effects of ambient air temperature on heat loss from the human body and thermal comfort indexes were examined. With the increase in activity level, skin temperature covering active muscles was decreased. Similarly, the heat loss from the body reduced with decreasing core temperature under the same conditions. With the rising activity level, increasing body temperature was compensated with decreasing skin temperature. At the low ambient temperature, PMV took negative values. If air temperature rose, PMV was positive. At high and low ambient temperature, heat balance between human body and environment was not established. Therefore, these conditions had negative impacts on human thermal comfort relatively.

On the other hand, Rating of Perceived Exertion (RPE) is an index, commonly called the Borg Scale, which is a well-known and widely used tool to evaluate subjective feeling of fatigue [9]. The RPE scale measures the perceived exertion during exercise based on the sensation of the participant individually. Generally, it consists of 15 steps ranging from 6 to 20, together with the performance level and the exercise intensity can be checked easily by subjective rating. It starts from “very very light” at the scale 6, reaches to “hard” feeling at the scale 15 and “maximal exertion” at the end defined by the scale 20. This RPE scale is widely used by scientists and coaches as a tool for exercise prescription [10]. RPE value changes dynamically during exercise. For example, athletes can set the exercise intensity to 15–16 RPE. With this point, it can be regulated by self-selected exercise intensity. It has been shown that RPE responses are not affected by gender status and not comparable during different exercise type [11]. Benefits of using RPE scale during exercise is to obtain information about fitness levels or it may be used for test termination criterion [12]. Additionally, it was shown that increases in RPE values in hot environmental conditions are bigger than cool condition, relatively at the same work output. Even though the intensity of exercise decreases or remains constant in these conditions, the brain perceives that the exercise is getting gradually harder [13,14]. Additionally, Kenny et al. examined the effects of different exercise modalities in the cool and cold environments on RPE responses. They showed that the use of RPE may not be appropriate for reflecting the total work of exercise if there are other stressful factors beside the exercise [15]. This indicates that the RPE is more effective tool in the cool condition than hot condition. Celine et al. compared heart rate and RPE based on the training program over 6 weeks and both provided same physiological adaptation [16]. This indicates that RPE may be an important parameter that can be used for exercise load. Kenny et al. investigated sensible and latent heat loss and the heat storage of human body [17]. These values were measured via direct and indirect calorimeter simultaneously, and the exercise conditions were evaluated by RPE. Schlader et al. also investigated RPE values during exercise at warm and cold environmental conditions [18]. Crewe et al. examined RPE values of seven cyclists who performed at different work outputs in the warm and cold environmental conditions, relatively [13]. Tucker et al. investigated the human thermal behaviour during exercise for various temperatures at a fixed RPE value [14]. In a recent study, skin temperature data obtained by infrared thermography of athletes at sub-maximal exercise loads revealed that skin temperature decreased until the 8th minute of exercise and then gradually increased [19]. The skin temperature responses were lower in maximal exercise compared to submaximal exercise.

There are a lot of studies about PMV and RPE separately in the literature. There are also a few studies about thermal comfort during exercise and exercise conditions [4,20]. However, there is no study on the relation between PMV and RPE within the knowledge of the authors. The aims of the research are to analyse a relationship between PMV and RPE during exercise and to observe the relationship to differ between highly trained and less trained athletes. In this study, we hypothesized that PMV and RPE closely related and this relation affected participants’ training status. For that purpose, the same experimental data with the referenced study [19] were analysed to investigate the physiological and thermal responses of the human body during exercise.

## 2. Materials and Methods

### 2.1. Climatic Test Chamber

**Figure 1:**
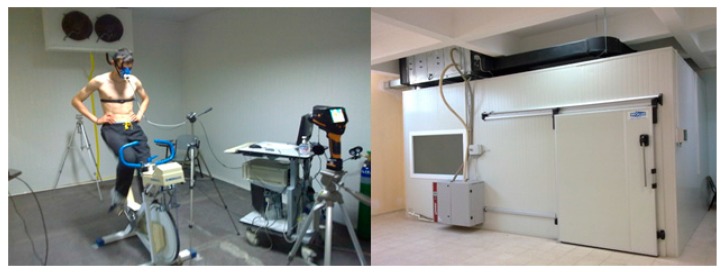
Outside and inside view of the test chamber.

The experimental studies were carried out in a test chamber at Faculty of Sport Sciences in Ege University. The dimensions of the test chamber are 6 m as length, 4 m as width, and 3 m as height (Figure 1). An electrical heater system included fan located at the top of the test chamber is shown in Figure 1. There are two electrical heaters whose capacity is 3 kW each and there are also two humidifiers inside of the system (the box front of the test chamber is the evaporative humidifier box connected with the electrical heater at the top of the test room in Figure 1). The energy recovery unit is also at the top of test chamber (the other side of the electrical heater system). Before inside air of the test, chamber is exhausted to the atmosphere, the energy of the exhaust air is recovered by fresh air in the heat exchanger of the energy recovery unit. Air circulation is conducted via four vent holes. Two intake ducts of vent hole are connected with the heat recovery system and the electrical heater system, separately. On the other hand, one vent hole is the exhaust vent of the heat recovery system. The other vent hole is the exhaust vent of the electrical heater system. All procedures of the experimental studies were performed under the condition of 21.3 ± 0.4 °C temperature and 64.5 ± 2.5% relative humidity in the test chamber equipped with the ability to control the inside temperature and the humidity.

### 2.2. Participants

The study was designed according to the rules and principals of Helsinki Declaration protocol and approved by the university ethics committee (EGE.ETK.09.09-3/18). Eleven participants volunteered for the study. The experiments were carried out with 11 subjects in a conditioned test chamber by using a cycle ergometer. Participants were experienced in athletic training and had participated in formal exercise training for five years. They had no smoking, alcohol or other substance addiction. They did not have any systemic disease and were disability free. They have not been taking any legal or illegal drugs which may affect physical performance and human metabolism. Experiments were carried out at the same time every day to observe chances in circadian rhythm. Test procedure was read and signed by each subject individually and tests were carried out according to the same standards.

### 2.3. Protocols

Two familiarisation sessions were carried out to adapt the test chamber. One or two days after familiarisation sessions, all participants’ anthropometric data were measured. Then, a graded submaximal exercise test was performed which consisted at least four 5-min stages and the procedure was continued until the respiratory threshold was reached. After 30–45 min. of the graded submaximal exercise test, a graded maximal oxygen consumption (VO_2max_) tests were performed to determine the aerobic power of each subject individually. Initial load of VO_2max_ test was set respiratory threshold which reached in the graded submaximal exercise test. Stage durations in the graded maximal oxygen consumption test were set at one 4-min and three 2-min phases, followed by 1-min phases via 27–36 W load increment at each stage in the cycle ergometer. Fixed cadence was 90 rpm through the test. Then, a constant load submaximal exercise test was performed with wattage corresponding to 60% of VO_2max_ level for 20-min in a different day. 60% of VO_2max_ level was calculated by using graded submaximal exercise test result. Totally, the subjects were called to the test chamber for four days.

### 2.4. Thermal Data Measurements

Skin temperature measurements of the participants were executed by a thermal camera (Testo 875-1 ThermaCAM, Frankfurt, Germany). Chest and back regions thermal images were analysed using an area according to the reference points [12]. Each area was evaluated with 160 × 120 pixel resolution and 0.08 °C thermal sensitivity. Emissivity value of the subject’s skin was set as 0.98 [21] in the thermal camera which had been calibrated in the manufacturer’s laboratory. On the other hand, the core temperature was measured by VitalSense^®^ which transmits data continuously to the data logger by using an ingestible telemetric temperature sensor (Mini Mitter Co., Inc., Bend, OR, USA). The volunteers took temperature pills one hour before their experiments [19]. Temperature and humidity of the indoor environment of the test chamber were controlled and recorded during the experiments. The measured and calibrated data for the constant load submaximal tests were considered in the presented study. Also, PMV values were calculated by using definitions mentioned in ASHRAE [3].

### 2.5. Statistical Analysis

Results were evaluated by using SPSS 20.0 (SPSS Inc., Chicago, IL, USA) statistical software. Descriptive results were reported as mean values and standard deviations (±SD). Differences were calculated as percent differences of mean absolute values. After Skewness and Kurtosis, Shapiro-Wilk test of normality was used to determine whether the distribution of values is normal or not. Spearmen method was used for the correlation analysis [22]. It was regarded that the number of divided groups fit for the correlation analysis [23,24].

## 3. Results

**Figure 2:**
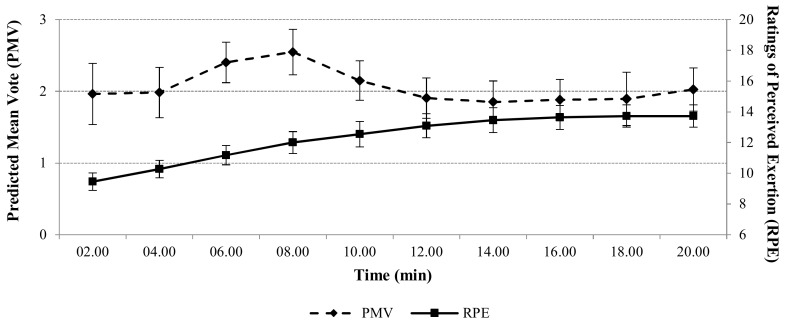
Average Predicted Mean Vote (PMV) and Rating of Perceived Exertion (RPE) responses of the subjects during the constant load submaximal test sessions.

The average RPE value started from 6 at the beginning of the exercise and then increased throughout the test and after 14 min of the exercise remained constant as seen in Figure 2. The mean value of RPE was 12.3 ± 2.36. Average PMV value started from 1.96 at the beginning of the exercise and then increased until the eighth minute in the sequel slightly decreased and remain constant until the end of the exercise (Figure 2). The mean value of PMV was calculated as 2.06 ± 1.06.

### 3.1. First Step: PMV-RPE Correlations

The average correlation coefficient between PMV and RPE was calculated to be −0.51 and the level of correlation coefficient was estimated to be a moderate level as shown in Table 1. The correlation coefficients were low at the first-time steps. At the rest of the time steps, the correlation coefficients observed higher at the second half of exercise.

### 3.2. Second Step: PMV-RPE Correlations at Different Oxygen Consumption Rates

The subjects were divided into two groups according to the amount of oxygen consumption. The oxygen consumption of four subjects were significantly larger than the average value which was 54 mL⋅kg^−1^⋅min^−1^. Therefore, the high oxygen consumption cohort consists of four subjects while low oxygen consumption cohort consists of seven subjects. The correlation between PMV and RPE at high oxygen consumption rate was −0.21 as shown in Table 2. The correlation coefficients were very low initially and became higher at the second half of the exercise but the average of correlation coefficients was relatively weak. However, a moderately strong correlation coefficient was calculated for low maximal oxygen consumption cohort (*r*: −0.77). The correlation coefficient started from the moderate level of relation and increased to the strong level. The correlation coefficient was differentiated into strong level from moderate level (Table 2).

## 4. Discussion

In this study, the relationship between RPE which measures the physiological stress and PMV which measures the thermal comfort was investigated. It was shown that the relation between RPE and PMV was −0.51 in our study cohort while this correlation increased to −0.77 for the moderately trained and recreationally active participants instead of the highly trained cohort.

The thermoregulatory system in the human body was activated and tried to be established the thermal equilibrium with the environment. Nevertheless, the rate of thermal discomfort is higher than the neutral value of the thermal comfort during the exercise. The physical activity leads to discomfort in the human body depending on the exercise intensity and duration. Revel and Arnesano [20] studied the thermal comfort conditions and the energy consumption by using PMV scale. The authors have presented PMV as a necessary tool by using dynamic measurements for the gym, fitness salon, and pool. According to abovementioned study, the thermal conditions of the swimmers whose skins were dried, felt slightly warm and their average PMV values were approximately 1.25. However, the condition for the swimmers whose skins remain wet, felt slightly cool with respect to the thermal comfort condition [20]. Vanos et al. [25] evaluated exercise conditions in the outdoors for improvement recreation region. Conditions were analysed with regard to the formula of COMfort^®^ energy balance model. In this current study, PMV values remained constant after a low increment which was caused by steady heat production due to relatively low exercise intensity. Furthermore, PMV value of some subjects increased up to 3.5 which are higher than the original PMV scale range from −3 to +3. It can be argued that a new formula can be developed to evaluate the thermal comfort index for different exercise conditions and RPE level can support to create an index for estimating participant’s thermal condition level.

Crewe et al. [13] analysed RPE and estimated exercise duration by RPE rising. They carried out five experiments at different conditions. During the experiments, RPE increased at constant work output and then they finished the trials when RPE value reached 20. Tucker et al. [14] investigated the exercise intensity at fixed RPE rates at different environmental conditions. They determined that the work output attenuates if participants continued to exercise at the fixed RPE value. Similarly, in this current study, mean RPE value was found as 12.3, however, it increased at the half of the exercise and increased up to 14 (Figure 2). Despite the constant work rate, the moderate increment in RPE, showed that the perceived exercise intensity became harder and participants slightly felt tired during the last period of exercise. Meanwhile, average RPE values almost settled at the last part of the exercise during the constant load.

After the relation between PMV and RPE was evaluated, the first two time intervals (at the second and fourth minute) were excluded from the dataset since the oxygen consumption of the participants did not reach the steady state condition. At the beginning of exercise intervention, the data were compared with respect to PMV and RPE each time interval. At the second stage, the data were divided into two groups with respect to the amount of oxygen consumption.

The correlation coefficients of PMV and RPE at low maximal oxygen consumption cohort were significantly higher compared with the high maximal oxygen consumption cohort (Table 2). It could be deduced that the aerobic performance status of participants affected the correlation between PMV and RPE values negatively which can also be caused by participants’ thermoregulation differences. It is known that high aerobic power athletes have a higher thermal regulation instead of lower ones [26,27].

## 5. Conclusions

In this study, thermal and physiological behaviours of human body were investigated experimentally during the exercise based on the correlation between PMV and RPE. It was showed that amount of oxygen consumption affected the correlation between PMV and RPE. Results showed that PMV and RPE relation ratio was higher in the low aerobic power group than those with the higher level group. Thus, it can be thought that the correlation coefficients between PMV and RPE decreased at high aerobic power level participants due to better thermal regulation.

It could be speculated that PMV might not reflect the real physiological stress of athletes during exercise. In other respects, PMV values of some subjects exceeded “3” at certain time intervals of exercise. Since the original PMV scale ranges from −3 to +3, a new formula can be developed to evaluate the thermal comfort index for exercise. RPE level can support to create an index for estimating the participant thermal condition level.

The investigation of PMV-RPE relation should be conducted with more subjects and short time intervals for measurements and different exercise intensities should also be evaluated for future studies.

## Figures and Tables

**Table 1:** PMV and RPE correlations during the constant load submaximal exercises (*n* = 11).

Time Interval	Corr. Coefficients	Level of Corr. Coefficients	*p* Values
04.00–06.00	−0.27	Weak	0.42
06.00–08.00	−0.26	Weak	0.44
08.00–10.00	−0.50	Moderate	0.12
10.00–12.00	−0.59	Moderate	0.06
12.00–14.00	−0.66	moderately strong	0.03
14.00–16.00	−0.65	moderately strong	0.03
16.00–18.00	−0.57	Moderate	0.07
18.00–20.00	−0.62	moderately strong	0.04
Average	−0.51	Moderate	

**Table 2:** Levels of PMV-RPE correlation for the high and low maximal oxygen consumption cohorts (*n* = 11).

Time Interval	High VO_2max_ Group Corr. Coefficients	Low VO_2max_ Group Corr. Coefficients
04.00–06.00	0.00	−0.49
06.00–08.00	0.40	−0.70
08.00–10.00	0.11	−0.84
10.00–12.00	−0.20	−0.75
12.00–14.00	−0.63	−0.95
14.00–16.00	−0.63	−0.81
16.00–18.00	−0.11	−0.82
18.00–20.00	−0.63	−0.77
Average	−0.21	−0.77
